# Assessment of the knowledge landscape, information needs and attitude towards decision support systems among hemp farmers in Florida

**DOI:** 10.1186/s42238-025-00318-3

**Published:** 2025-08-20

**Authors:** Alwin Hopf, Jonathan A. Watson, Mickie Swisher, Zachary Brym, Gerrit Hoogenboom

**Affiliations:** 1 Department of Agricultural and Biological Engineering, Institute of Food and Agricultural Sciences, University of Florida, Gainesville, FL 32611 USA; 2 Department of Family, Youth and Community Sciences, Institute of Food and Agricultural Sciences, University of Florida, Gainesville, FL 32611 USA; 3 Department of Agronomy, Tropical Research and Education Center, Institute of Food and Agricultural Sciences, University of Florida, Homestead, FL 33031 USA; 4 Global Food Systems Institute, Institute of Food and Agricultural Sciences, University of Florida, Gainesville, FL 32611 USA

**Keywords:** Industrial hemp, Technology adoption, Decision support systems, Farmer interviews

## Abstract

**Background:**

The recent legalization of industrial hemp (*Cannabis sativa L.*) in Florida and across the US has sparked interest among established farmers and newcomers alike. However, the nascent industry faces challenges due to limited location-specific cultivation knowledge, evolving regulations, and market uncertainties. Agriculture technology such as crop growth models and decision support systems (DSS) can support sustainable hemp production in new regions. However, the adoption of such technologies is limited and requires participatory work with and study of DSS users for the development of appropriate technology.

**Methods:**

This study explores the knowledge landscape, information needs, and attitudes towards decision support systems among hemp farmers in Florida through a series of semi-structured qualitative interviews.

**Results:**

We identified distinct farmer profiles, including established farmers seeking diversification, out-of-state hemp growers exploring Florida’s climate, hemp practitioners from non-agricultural backgrounds focused on quality, and first-time farmers driven by personal interest. Each profile exhibited unique motivations, information-seeking behaviors, and resource constraints. Agronomic challenges, such as pest and disease management, cultivar selection and time of planting were common concerns across all groups. Regulatory uncertainties and market volatility further compounded these challenges. While interest in DSS exists, particularly for addressing agronomic issues and optimizing decision-making, barriers such as cost, trust in model accuracy, and utility remain significant. Farmers expressed a preference for tailored, locally relevant DSS that offer actionable recommendations and integrate seamlessly into their existing workflows.

**Conclusion:**

The study underscores the importance of participatory DSS development, involving farmers in the design and validation process to ensure the tools meet their specific needs and build trust. Insights from this research will contribute to the ongoing development of a process-based crop growth model and DSS specifically designed for Florida’s hemp production.

**Supplementary Information:**

The online version contains supplementary material available at 10.1186/s42238-025-00318-3.

## Highlights

Decision Support Systems (DSS) have potential to support sustainable agriculture production, but experience low adoption rates among practitioners.

Study explored the knowledge landscape, information needs, and attitudes towards DSS among hemp farmers in Florida through semi-structured interviews.

Distinct farmer profiles were identified that exhibited unique motivations, information-seeking behaviors, resource constraints and attitude towards DSS.

Interest in DSS exists particularly for addressing agronomic issues in absence of historic data and knowledge, but barriers such as cost, trust and utility remain significant.

Farmers expressed a preference for tailored, locally relevant DSS that offer actionable recommendations and integrate seamlessly into their existing workflows.

**Supplementary Information:**

The online version contains supplementary material available at 10.1186/s42238-025-00318-3.

## Introduction

### Hemp production in Florida

Industrial hemp (*Cannabis sativa L.*) is a versatile annual plant used for fiber, seed, oil, and medicine, with a history of cultivation dating back to 8,000 BC (Small 2015). Hemp fiber is considered a more sustainable alternative to cotton (Schumacher et al. 2020; Wise et al. 2023) and has various industrial applications, including as a potential bioenergy feedstock (Werf et al. 1996; Finnan and Styles 2013). Recent legalization has renewed interest in hemp in Florida and North America, prompting research into its production, economics, and social-ecological aspects (Cherney and Small 2016; Rampold et al. 2021).

Florida’s subtropical climate and diverse agriculture, along with the decline in citrus production (Her et al. 2017; Singerman and Rogers 2020), have spurred experimentation with new crops (Volk et al. 2017). Examples include hops, kenaf, blueberries, and more recently, pomegranates, ginger, and vanilla (Agehara et al. 2021; Joyner and Wilson 1967). This positions Florida with states like California or Hawaii in specialty crop experimentation, distinct from the Midwest’s focus on few commodity crops (Aguilar et al. 2015). While Florida produces 200–300 commodities (IFAS 2022), a general decline in US crop diversity and Florida farmland reduction due to urbanization are notable trends (Crossley et al. 2021; Volk et al. 2017).

In 2023, US outdoor hemp acreage (11,202 ha) and value (US$258.0 million) increased over 2022, but protected production (30.1 ha, US$32.9 million) decreased (USDA NASS 2024). In Florida, in 2023 only 19 and 16.2 ha were reported as planted and harvested respectively, a significant decrease from 2022 with 85 ha planted and 50.6 ha harvested and 2021 with 121.4 ha planted and 66.8 ha harvested. The area under protected production in Florida showed a similar decline with 12,213 m^2^ (1.2 ha) in 2023, 65,484 m^2^ (6.5 ha) in 2022 and 92,050 m^2^ (9.2 ha) in 2021. These numbers indicate an early saturation of indoor hemp production for flower and essential oils, which dominated the early years of the “hemp boom” in the US. The future economic prospects for open-field hemp in the US and Florida remain uncertain (Mark et al. 2020). Key challenges for Florida hemp include inconsistent regulations, need for better varieties and practices, and supply-chain bottlenecks (Blare et al. 2022). Limited knowledge on hemp production and economics is a nationwide issue (Adesina et al. 2020). Knowledge mapping (Aryal et al. 2023) and further study on Florida farmers’ information needs are essential for developing a sustainable hemp industry.

### Knowledge landscape and information needs

Farming in the US is an enterprise deeply rooted in knowledge derived from a complex and multifaceted landscape of information sources. Understanding how farmers find, evaluate, and utilize information is crucial in supporting technology development, promoting sustainable practices, and fostering innovation in the agricultural sector (McArthur Jr 2001; Šūmane et al. 2018).

A range of formal and informal sources of knowledge are used by farmers (Beethem et al. 2023; Houser et al. 2019; Velandia et al. 2021; Wójcik et al. 2019) (Table 1). Demographics, production systems, and trust in the credibility of the source of information are factors influencing the information seeking behavior of farmers: A farmers age, education, and farm size can influence their preferred information sources and adoption of new technologies (Isgin et al. 2008; Martínez-García et al. 2014). The type of crops grown, preference for organic practices versus conventional methods, or niche markets can influence the specific knowledge farmers seek (Charatsaria et al., 2012; Morgan and Murdoch 2000). Generally, farmers place high value on the reliability, validity, and practical relevance of information (Jørgensen et al. 2007; Rose et al. 2016; McCown 2002a,b).

**Table 1:** Formal and informal knowledge sources of farmers in the US (Beethem et al. 2023; houser et al. 2019; Velandia et al. 2021; Wójcik et al. 2019)

Source	Description
Land-grant Universities and Cooperative Extension Systems	Conduct research on best practices, crop varieties, pest control, and emerging technologies. Disseminate this knowledge through publications, field days, workshops, and personalized consultations.
Government Agencies	The United States Department of Agriculture (USDA) and its various agencies like the National Agricultural Statistics Service (NASS) and the Farm Service Agency (FSA) provide a wealth of resources. Farmers rely on their data for market reports, crop insurance, weather forecasts, and policy updates.
Supplier Relationships	Seed, fertilizer, and equipment suppliers are important sources of information for farmers. Sales representatives and technical support staff provide product knowledge, application advice, and troubleshooting assistance for specific products or management aspects.
Consultants	Farmers often seek specialized advice from independent consultants who offer expertise in areas such as pest management, fertilizer recommendations, or precision agriculture technologies.
Trade Publications	Trade publications and industry websites provide news, market analysis, and technical articles relevant to specific crops or farming practices.
Peer Networks	Farmers interact with neighbors, attend conferences, and participate in local organizations to share experiences, learn from each other, and find solutions to common challenges.
Online Forums and Social Media	Farmers increasingly utilize online platforms to connect with peers across the country, access information quickly, and engage in discussions about timely issues and emerging technologies.

The US farmers’ information landscape is constantly evolving due to market and technological advancements. Farmers, especially on highly mechanized, large-scale farms, increasingly use data from sensors, drones, and management software (Asseng and Asche 2019; Lowenberg-DeBoer and Erickson 2019; Karunathilake et al. 2023). The ability to interpret this data is vital for farmers and other agricultural stakeholders.

Consumer demand for local food, supply chain transparency, and animal welfare requires farmers to communicate their practices (Astill et al. 2019; Bastian and Zentes 2013). Concurrently, a changing policy and environmental scene, emphasizing sustainability—including soil health, water quality, and climate-smart agriculture—drives demand for new knowledge (Bucci et al. 2018; Hazell and Wood 2008). Research must understand this shifting landscape to effectively support farmers.

Understanding farmers’ information navigation is crucial for agricultural value-chain actors. Technology developers design tools to integrate with existing knowledge and solve production challenges. Extension providers tailor educational programs to farmers’ preferred channels and concerns (Carroll et al. 2022), while policymakers must recognize diverse information needs to foster a knowledge-driven, innovative agricultural sector.

The US farmers’ knowledge landscape is as varied as the farmers, with research addressing specific demographics like small or urban farmers (Goodwin and Gouldthorpe 2013; Campbell et al. 2022) or a broader range of stakeholders (Jagtap et al. 2002). It should also be noted that some information or sources are not directly consumed by farmers, but rather mediated via extension agents which can then customize and localize the information (Baldin et al. 2021; Beethem et al. 2023). This intermediary role is vital as farmers possess diverse operational goals—such as prioritizing profitability, sustainability, risk reduction, or labor efficiency—which influences their receptiveness to, and requirements for, decision support system (DSS) tools. Advisors can help tailor or interpret DSS outputs to align with these varied individual farm priorities (Ayre et al. 2019; Eastwood et al. 2019). Appreciating this complexity and adapting to emerging trends can help create DSS that empower Florida’s farmers to innovate.

### Decision making

**Fig. 1:**
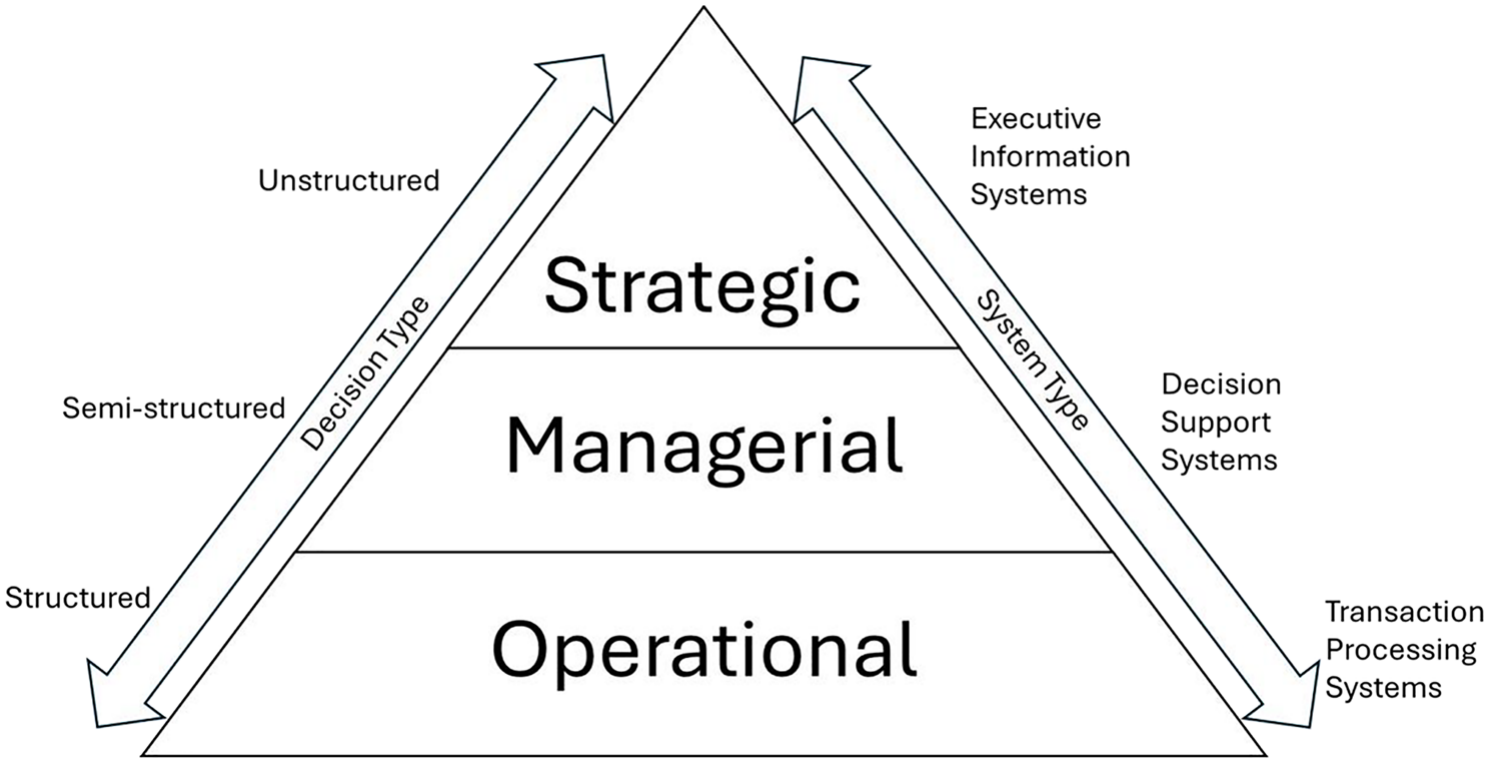
Decision and system type pyramid adapted from Roch et al. (2022) under CC BY-NC-SA 4.0 license

Farms and agribusinesses face complex decisions. Categorizing them can streamline processes and improve farming efficiency. A traditional model (Fig. 1) outlines three decision levels: (1) Strategic decisions, by top management, address long-term goals and overall farm direction. Often unstructured, they involve ambiguous criteria and incomplete information, requiring creativity, intuition, and judgment. Technology can aid information gathering. (2) Managerial decisions, by middle managers, translate strategic goals into actionable plans. These can be structured, semi-structured (combining known factors with human judgment), or unstructured, depending on the situation. (3) Operational decisions, made by field employees for day-to-day tasks, are typically fully structured: repetitive, with clear rules, based on known inputs, and suited for automation.

Decision Support Systems (DSS) are designed for semi-structured managerial decisions, but agricultural decision-making, especially on smaller farms, often lacks this clear structure. Decision layers may overlap, with one person handling multiple roles. Research by Öhlmér et al. (1998) detailed decision event stages. Human decision-making is complex, dynamic, and influenced by both quantitative (financial, efficiency) and qualitative factors (emotions, preferences), which can cause inefficiencies (Brehmer 1992; Taramuel-Taramuel et al. 2023). Models of farmer decision-making show influences from socio-demographic, psychological, household, farm, and social factors (Edwards-Jones 2006). DSS support, but often do not make decisions (Silver 1991). While AI and autonomous systems could redefine decision-making (Holzinger et al. 2022; Lai et al. 2023), this study did not focus on them due to the early stage of Florida’s hemp industry.

### Agriculture decision support systems

**Fig. 2:**
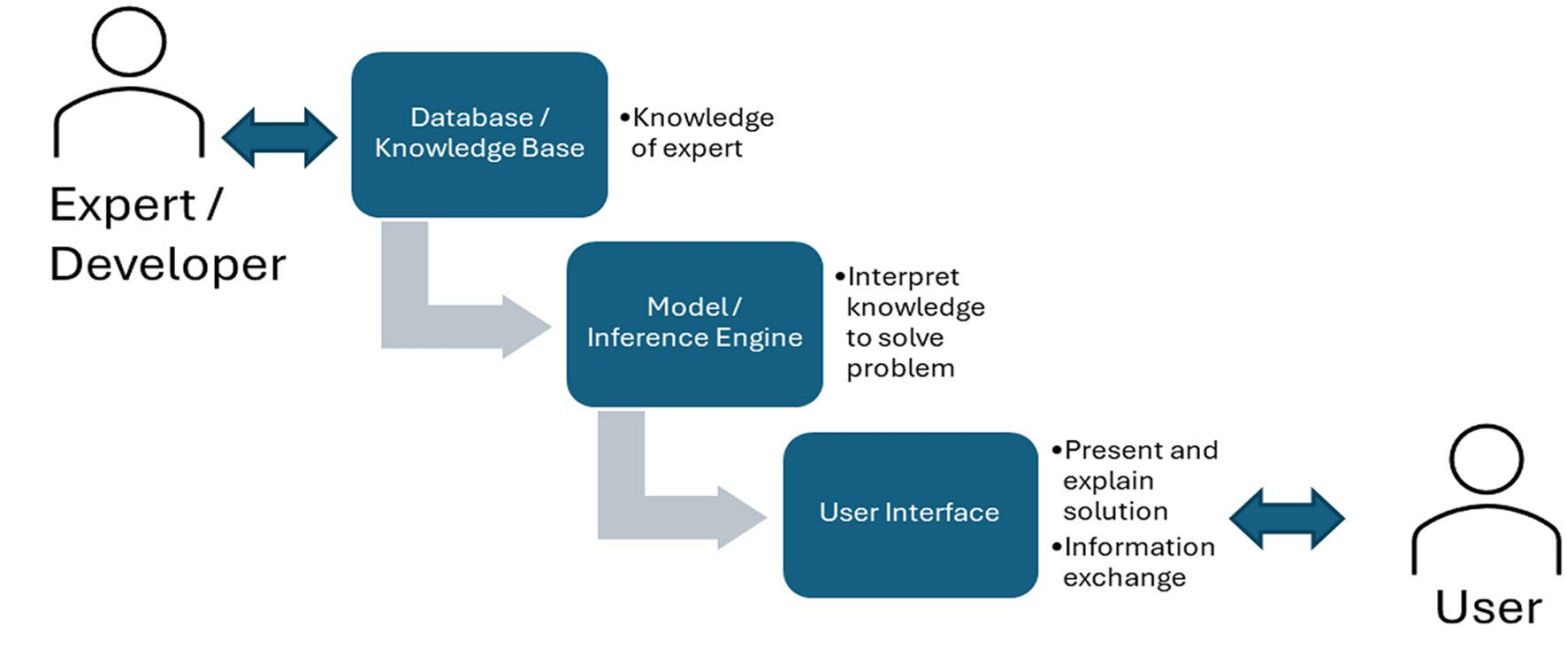
Decision support system schemata connecting an expert developer with a user. (source: main author)

Originating from organizational decision-making studies in operations research, Decision Support Systems (DSS) are found across many domains and are often designed for non-technical users (Power 2002). Key components include a database/knowledge base (aggregating information for context), an inference engine/model (applying rules for decisions), and a user interface (Fig. 2).

DSS layouts vary by use case (e.g., diagnostic, advisory, control) (Manos et al. 2004), employing diverse models from statistical tools for irrigation (Torres-Sanchez et al. 2020) to crop simulations for fertility (Kihara et al. 2012) or machine learning for soil mapping (Gasmi et al. 2022). These models use data to generate forecasts, evaluations, and recommendations. The user interface is vital for interaction, allowing users to query the system, receive advice, visualize data, customize settings, explore scenarios, and manage data input.

Agricultural DSS integrate diverse data and models to guide tactical decisions in areas like crop management, resource allocation, and risk assessment. They provide data-driven insights for optimizing operations, improving productivity, and sustainability. Specific uses include managing irrigation (Ara et al. 2021), fertilizers (Villalobos et al. 2020), pests (Damos 2015), weather risks (Fraisse et al. 2006), and informing policy (Bazzani 2005).

DSS are increasingly prominent due to precision agriculture advances and the need for informed decisions in a complex agricultural landscape, often seen as key to Agriculture 4.0’s system of systems (Zhai et al. 2020). However, their limited adoption by practitioners has spurred theories on digital farming technology uptake.

Technology adoption involves successfully integrating and using new technology. While various theories (Wisdom et al. 2014) explain innovation adoption generally, they don’t specifically target agricultural innovation. Two prominent models, the Diffusion of Innovation Model (Rogers et al. 1962) and the Technology Acceptance Model (TAM) (Davis 1989), are often used to understand acceptance and adoption in agriculture.

The Diffusion of Innovation (Table 2) model explains how innovations spread within a social system. It highlights four key elements: the innovation itself, communication channels, the social context, and the time taken for diffusion. People are categorized from innovators to laggards based on when they adopt, and their perception of an innovation’s characteristics (e.g., relative advantage, complexity) influences this. The adoption process involves five stages: awareness, interest/need, evaluation, trial, and finally, adoption and continued use.

**Table 2:** Overview and definition of constructs and dimensions of the ’innovation of diffusion’ model (Rogers et al. 1962)

Construct	Dimensions	Definition
Innovation it-self, ease of adoption	Relative advantage, compatibility, complexity, trialability, observability	Attributes of innovation, each of them can promote or hinder the adoption of innovation
Communication	Direct communication, observation of peers, mass media	Means and mechanisms by which an innovation is passed from individual to individual
Social system	Context, culture and environment	Social norms and structures that affect how an innovation passes through a population
Time	Adopter groups from early to late adopter	Relative amount of time an individual takes to adopt innovation. Groups have common personalities, socioeconomic status, and communication

The Technology Acceptance Model (TAM), originated by Davis (1989) and later extended, explains technology acceptance through perceived usefulness and ease-of-use. Subsequent versions incorporated trust, perceived risk, and interventions to boost adoption, especially for e-commerce and digital technologies. TAM posits that various determinants influence perceived usefulness and ease-of-use, shaping a behavioral intention to adopt, which is further affected by subjective norms, ultimately leading to adoption or non-adoption. While TAM 3 (Venkatesh and Bala 2008) was considered more beneficial for this study due to its digital technology focus and potential as an informative framework, the actual TAM was not used in the study design or for quantitative evaluation due to limitations in the target population and sample size Table 3.

**Table 3:** Overview and definition of constructs and dimensions of the ’technology acceptance model’ model (Davis 1989)

Construct	Dimensions	Definition
Individuals’ perception of technology	Perceived ease of use, perceived usefulness	Effort necessary to use/adopt technology Benefits from adopting technology
Environment	Norm, image, job relevance, output quality and outputs demonstrability	Outside factors, environmental and social norms influencing individuals’ decision
Attitude	Self-efficacy, perception of external control, anxiety, playfulness, perceived enjoyment and usability	General attitude towards digital technologies, e.g., computer-based information system

To prepare this study, decision-making models informing Technology Acceptance Model (TAM) were reviewed, including the Theory of Reasoned Action (Fishbein and Ajzen 1977), which links intention (via attitude and norms) to behavior; the Theory of Planned Behavior (Ajzen 1991), which adds perceived control; and Social Cognitive Theory (Bandura 1999), noting interactions of personal, environmental, and behavioral elements. The Diffusion of Innovation Model was also considered, but its temporal adoption aspects, socio-economic factors, and social system dynamics were less central. This research aimed not for quantitative adoption models for precision agriculture or decision-support tools (DSS), but for practical guidance to refine DSS for grower challenges. This social science component informs future engineering. Consequently, TAM’s constructs of perceived usefulness and ease of use were deemed most beneficial for this research.

A large body of knowledge can be found when narrowing down from general innovation to a focus on agriculture technology and innovation. Agricultural technology, converting inputs to outputs (Foster and Rosenzweig 2010), includes digital innovations where adoption is gauged by purchase and use intensity (Bolfe et al. 2020; Gabriel and Gandorfer 2023). Farm characteristics, policies, and regulations are key adoption factors (Griffin and Lowenberg-DeBoer 2005; Lowenberg-DeBoer and Erickson 2019; Lowenberg-DeBoer et al. 2021).

Previous research (Zhai et al. 2020) identified critical issues in agricultural DSS: functionality, requirement analysis, user interfaces, and user consideration. Farmers may struggle with confusing interfaces. Developers sometimes overlook farmers’ specific needs, creating misaligned systems. Current DSS are often narrowly specialized, lacking integration and holistic understanding of factors like climate or soil, leading to flawed advice.

Engineering barriers for internet-based precision agriculture tools and DSS include cost, power, connectivity, and data-related challenges (Hundal et al. 2023). For pest management DSS, perceived risk is a key adoption barrier due to potential yield impacts (Gent et al. 2011). Generally, farmers expect clear recommendations for optimal decision-making from DSS, with little tolerance for incorrect recommendations or suboptimal results (Brugler et al. 2023; Debeljak et al. 2019; Rose and Bruce 2017). Social norms and networks also influence farmer decision-making (Burlig and Stevens 2019, 2023; Hüttel et al. 2022).

Modeling agricultural technology adoption is an active research area (Montes de Oca Munguia et al., 2021), with examples like combined empirical and agent-based models (Shang et al. 2021). However, unlike natural sciences, process-based modeling of adoption and human decision-making in social sciences involves numerous coexisting theories with divergent assumptions (Groeneveld et al. 2017). The review of these theories and case-studies concluded that agricultural DSS can empower farmers with data-driven insights, but traditional top-down development has limited adoption by inadequately addressing farmer diversity (McCown 2002a, b). Conventional DSS research often overemphasizes technical aspects (Courtney 2001), needing more user consideration and ‘design science’ (Arnott and Pervan 2005, 2014). Participatory DSS development, engaging stakeholders throughout, is crucial for creating solutions aligned with farmers’ real-world needs, improving technology integration and adoption.

### Participatory development of decision support systems

Incorporating farmers in research, or ‘participatory action research’ (Vaughn and Jacquez 2020), aims to improve research outputs and outcomes through methods like needs assessments, training, evaluations, and on-farm trials. Key principles involve farmers as co-creators contributing practical knowledge alongside expert scientific input (Oliver et al. 2012). This participatory approach benefits researchers by refining questions and methods (Vaughn and Jacquez 2020), yielding more relevant, reliable Decision Support Systems (DSS), and fostering trust and participant ownership.

Building on this, Jakku and Thorburn (2010) highlighted social learning in participatory DSS development, valuing the learning process itself. The GOSSYM/COMAX cotton management system (McKinion et al. 1989) exemplifies this: users gained significant knowledge (e.g., on nitrogen management), leading to internalized understanding and reduced DSS use (Albers 1990). This points to an extended definition of ‘technology adoption,’ where declining DSS use can indicate successful adoption if its heuristics are internalized by users (Ara et al. 2021).

Despite advantages, participatory DSS development faces challenges such as increased demands on time, finances, and skilled facilitation, alongside difficulties in managing diverse stakeholder groups and potential power imbalances (Van de Fliert and Braun 2002; Zhai et al. 2020). Nevertheless, most literature concludes that it is a worthwhile and urgently needed endeavor to deliver on research claims (Ashby and Sperling 1995; Cerf et al. 2012; Hoffmann et al. 2007).

### Problem statement

Linking back to industrial hemp and agronomic challenges, understanding the variability and interaction of genetic, environmental and management factors is a critical step during the introduction of a new crop or to develop locally adapted cultivars and management approaches (Hatfield and Walthall 2015). Given the complexity of these interactions, data science and process-based crop growth modeling is a promising approach to advance our understanding of the hemp crop itself and the surrounding agriculture system (Jones et al. 2017). At the same time, hemp as a new crop is attracting a diverse audience of potential growers, both experienced and new to agriculture production. A DSS can collate hemp growth models and various data sources to help study hemp production systems by evaluating cultivars, forecasting production under different growing conditions, and comparing management alternatives.

**Fig. 3:**
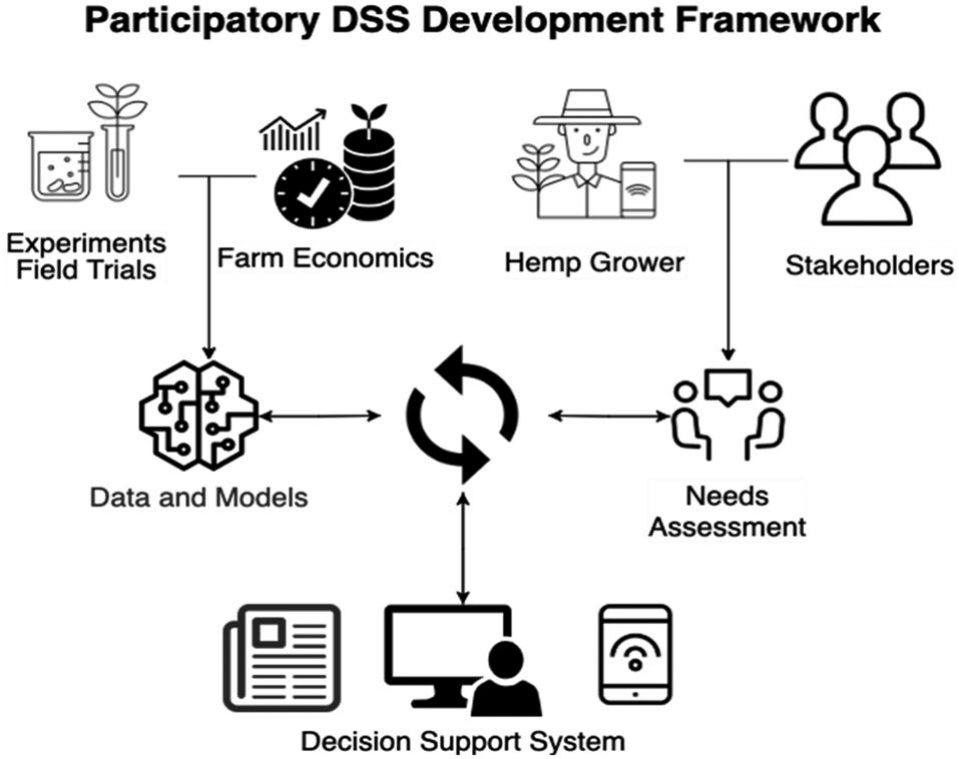
Participatory decision support system development framework. (source: main author)

Including participatory components into the development of this DSS can ensure that the tools and processes remain valuable for different types of users (Fig. 3). Addressing the current agronomic challenges through supportive policies, infrastructure investments, ongoing research, and public education is critical to unlocking the full potential of hemp. If successful, hemp production could become a valuable new crop for Florida agriculture, creating jobs, boosting economic development, and providing consumers with sustainable, locally sourced products.

### Objectives

The goal of this research was to inform the development of a process-based crop growth model and decision support system for industrial hemp production in Florida through an exploratory series of semi-structured interviews. The specific objectives were to (1) map the knowledge and information landscape of hemp farmers, (2) assess farmer profiles and the agronomic and management challenges of hemp growers and (3) discuss their attitude towards and needs for digital advisory tools and model-based decision support systems.

## Materials and methods

### Study design

This study utilized a qualitative research design with semi-structured interviews. This approach allowed for in-depth exploration of the farmers’ experiences, knowledge, and perspectives on the topics of hemp, agronomic challenges and DSS. The overall study was designed to have high information power with a narrow aim and high specificity, meaning that the sample creates more information with a smaller number of participants. The study design was chosen to allow the analysis of individual cases and extensive dialogue with participants. A comprehensive interview guide was developed based on the research objectives and through a review of relevant literature (Adams 2015; Magaldi and Berler 2020; Dearnley 2005). The interview protocol was informed from the underlying theories of Innovation Diffusion and the Technology Acceptance Model (TAM).

Based on these theories and the literature, several theoretical constructs were defined to guide the interview guide development and subsequent data analysis. The guide covered topics on.

(a) knowledge and experience with hemp cultivation practices, (b) awareness of regulations and best management practices for hemp farming, (c) information needs and preferred information sources and (d) experience with and attitudes towards DSS in agriculture. If applicable, additional questions were asked on the perceived usefulness of DSS for hemp farming, willingness to adopt DSS and the understanding of different methodologies behind DSS, such as AI, machine learning, process-based models or linear regressions.

The questions were kept general without a specific focus on either horticultural flower hemp for phytocannabinoids or agricultural fiber and seed hemp. Both types of production were expected to be present in the study sample and the interview guide was targeted at distilling more overarching concepts applicable to both. The semi-structured format provided a flexible framework that was guided but not limited by the prepared interview questions. Participants had the freedom to divert from structured questions, for example via probing follow-up questions to capture ad-hoc information (Bryman 2016; Warren 2012). This improved the theoretical rigor and explanatory power of the study’s conclusions.

The study was approved as IRB exempt based on the University of Florida’s IRB determination tool (https://research.ufl.edu/research-operations-services/secure/autotool.html, Submission ID: 16950). An expert review and cognitive test with five experienced farmers and survey practitioners was conducted before the interviews. The goal was to test the interview guide for clarity and to verify that the questions produce the desired type of answers. Questions were refined or reworded as necessary. The full interview guide and recruitment materials are presented in Additional Files A, B and C. No quantitative or socio-demographic data was collected as part of the study due to the small sample size (*n* = 15) and resulting challenges for generalization and validity of quantitative results.

### Study area and sampling

Purposive sampling was used to recruit participants who have the characteristics necessary to provide meaningful insights for the research objectives. The target population was hemp farmers currently or previously operating in Florida with a minimum of one season of experience related to hemp cultivation. Individuals were identified through exchange with staff from the University of Florida’s Industrial Hemp program, extension services, referral from previous interviewees and a list of licensed hemp growers maintained by the Florida Department of Agriculture & Consumer Services. The latter provided a list of circa 700 individuals that were approved for hemp cultivation in Florida, although the statewide reported harvest acreage indicated that only circa 10–20% of individuals on this list cultivated hemp.

The inclusion criteria were that individuals are currently involved in hemp farming in Florida or were in the past for a full season since the legalization in 2019. Involvement in hemp farming was defined as direct cultivation of the plant but also experiences from working in up-stream or down-stream sectors of the industrial hemp value chain were considered if they are closely linked to the cultivation of hemp. This included, for example breeding, farm equipment suppliers, cooperative extension services and contract farming schemes as well as processing or marketing activities for industrial hemp in Florida. Furthermore, individuals had to be willing to participate in a recorded interview without compensation provided. The overall sample was not exhaustive, since not all hemp farmers in Florida were reached.

### Data collection

After circulation of the interview materials, a total of 15 interviews were conducted from January to June 2023. Interviews were conducted face-to-face, or virtually via telephone or a video call according to the participants’ preference considering scheduling and distance. Each interview was audio-recorded with the participant’s permission and lasted 20–90 min, with most interviews lasting approximately 45 min. All audio recordings were transcribed verbatim using speech-to-text software and manual editing. The transcripts and interview notes were190 anonymized.

### Data analysis

The transcribed interviews were coded and analyzed with the assistance of the qualitative data analysis software, QSR NVivo Version 12 Plus (QSR International, 2018). Thematic analysis was conducted to identify recurring themes and patterns within the interview data, following examples of Castleberry and Nolen (2018), Mishra and Dey (2022) and Welsh (2002). Thematic analysis is conducted by closely examining qualitative data, like interview transcripts, to identify recurring patterns, ideas, and meanings, then organizing these patterns into “themes” that capture the core concepts emerging from the data. The analysis involves the steps of familiarization, coding, generating themes, reviewing themes, defining and naming themes, and finally writing up the results. This process involves reading and re-reading the data to identify key codes and then grouping those codes into broader themes that answer the research question (Castleberry and Nolen 2018; Mishra and Dey 2022; Welsh 2002).

## Results and discussion

### Thematic analysis

The inductive thematic analysis approach allowed for the emergence of key concepts, themes and codes related to the farmers’ knowledge landscape, information needs as well as attitudes towards DSS (Table 4). Throughout the analysis, comparative analysis was employed to ensure consistency and to refine the emerging themes (1) knowledge and networks, (2) resources and value and (3) trust and feedback. The analytical process and insights were documented with note-taking techniques. The complete analysis was conducted in multiple rounds, as additional codes and themes emerged or were condensed. Excerpt quotes (“…”) from interviews are presented in the text to support the results.

**Table 4:** Themes, codes and exemplary excerpts used for coding and analysis of interviews. Quotes from interviews indicated by parentheses (“…”). Explanations were added to some quoted interview excerpts in brackets [] for context

Theme	Code	Excerpt
Knowledge and Networks	Information Source	“the best information comes from outside Florida”, “there is a lot of ’broscience’ [science with questionable credibility] out there”, “the vendors will sell you anything, but they don’t have experience in Florida”, “we are in frequent exchange with the university extension”
	Peer Exchange	“we get together and trade-shows or events to exchange”, “others did not wanted to share”
	Motivation	“we are always looking for new crops to grow”, “we are third generation growers of …, hemp was something new and exciting”, “I have a personal and family connection to the plant”
	Knowledge Transfer	“I was completely new to farming”, “we knew how to handle the Florida climate from our vegetable operation”, “being so close to customers, I knew what was needed and tried to produce it”
Resources and Value	Cost of Decision Support Systems	“It takes too much time to learn a new software”, “software and hardware are quite expensive for a small operation as mine”, “the subscription costs the same for 10 or 10,000 plants”
	Actionable Recommendations	“don’t tell me what’s wrong, give me a solution”, “it would have been great to have a virtual tool to play around with before trying it out in the real field”, “I see using something like this to also study and understand some of our problems [early flowering, quality] in the past”
	Relative Importance of DSS	“for hemp, there are too many other aspects to get right first before looking at sensors and software”, “we need better genetics first”, “at the end you still need to double-check everything and have people perform the tasks”
Trust and Feedback	Trust in Recommendations	“I would trust but verify the recommendation”, “there needs to be a mechanism to overwrite bad recommendations”, “at the end you still need to double-check everything”
	Computer vs. Human	“I think AI, with the right data, is superior to human decision making”, “hemp is a special plant, not all of that can be captured in a software”, “unless there are robots you still need people to perform the recommended tasks”, “you get information from both types of interactions”

### Knowledge landscape and information needs

**Fig. 4:**
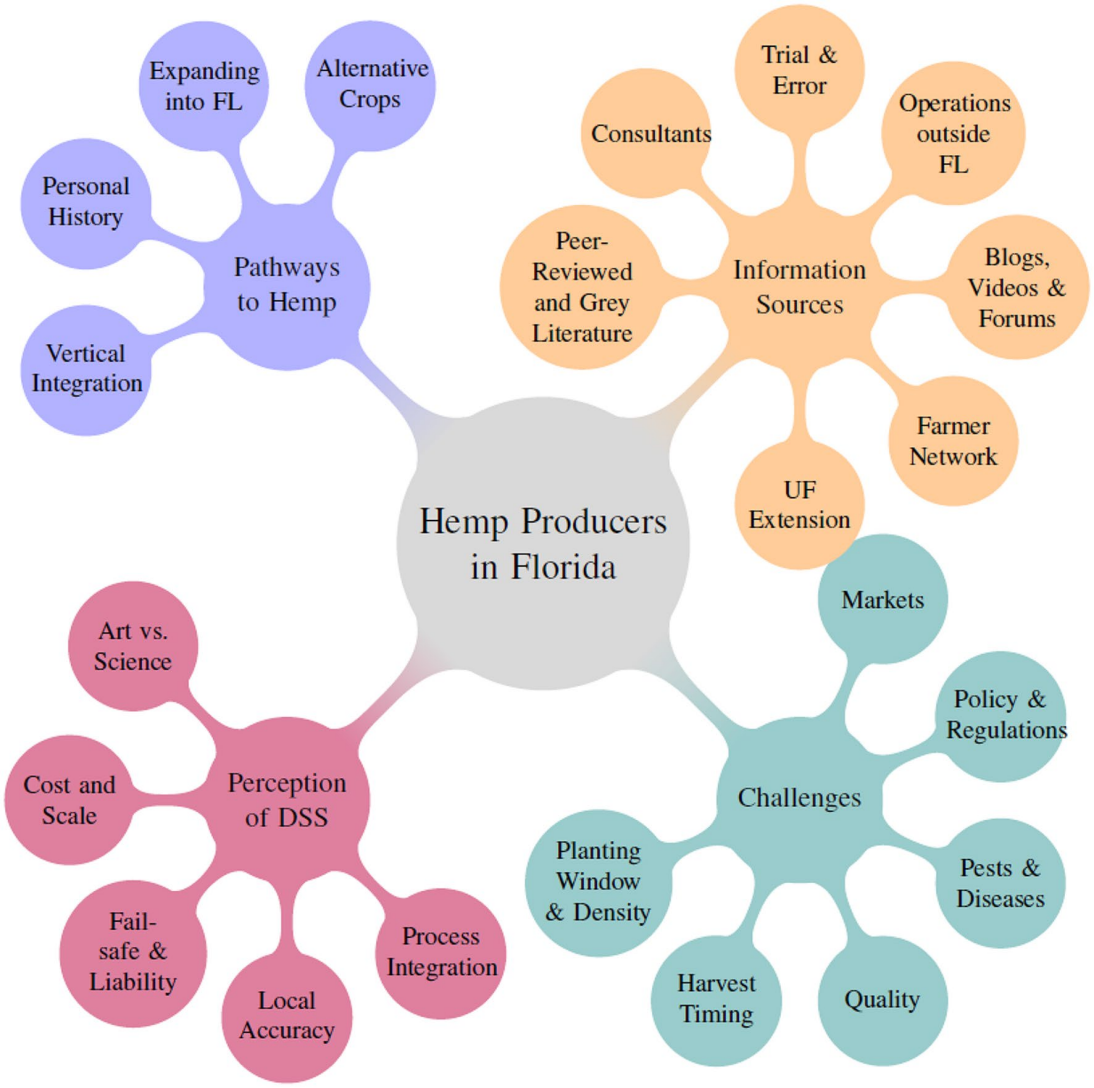
Information and knowledge landscape of hemp producers. (source: main author)

Drawing from the complete set of interviews, an overview of the information and knowledge landscape of hemp growers was derived (Fig. 4). It showcases the different pathways leading to hemp production, the information sources used, challenges encountered during production and the perception of DSS capacity to resolve some of these challenges. We subsequently grouped interviewees into distinct ‘farmer profiles’ for further analysis, as different interviewees varied in their responses and not all dimensions were relevant for each group.

### Farmer profiles

Interview results revealed a distinct grouping and heterogeneity in farmer profiles (Table 5). Groups of farmers differed in their profession, farming experience and general pathway and motivation that led them to hemp production. These farmer profiles have varying needs, preferences, and levels of expertise. Understanding these differences is important for developing a DSS that effectively supports these different potential users. Slightly more interviewees (*n* = 8) produced horticultural flower hemp for phytocannabinoids.

compared to fiber or seed hemp. This was due to the more promising market outlook and easier entry to production during the initial years after legalization in Florida. However, most interviewees discussed being open to producing any kind of hemp depending on market opportunities, which seem to have shifted towards grain and fiber hemp in recent years (USDA NASS 2024).

While this study sought overarching themes, the differing agronomic practices, production scales, and market dynamics between horticultural flower production and larger-scale fiber/seed operations likely influence their specific information needs and potential DSS applications. For example, flower producers might prioritize DSS modules for precise environmental control for cannabinoid profiles and pest/disease management in high-value, smaller scale systems, whereas fiber/seed producers may seek DSS support for optimizing planting densities, harvest timing for bulk biomass, and field-scale logistics. Although detailed distinctions were beyond the scope of these initial qualitative interviews, future DSS development for hemp should explicitly consider these segment-specific requirements to enhance tool utility and adoption across the diverse spectrum of hemp cultivation.

**Table 5:** Typical farmer profiles distilled from the interview series with different pathways to hemp production and characteristics

Profile	Pathway to Hemp production	Characteristics
Established Florida Farmer or Agribusiness (*n* = 5)	Income diversification, exciting new crop for farm succession planning, replace diminishing citrus production	Extensive resources and general farming experiences from existing operations, varying degrees of synergies with hemp production
Non-Florida Hemp Farmer or Agribusiness (*n* = 2)	Geographic diversification, extended growing season and market opportunities in Florida	Extensive resources and experience on Hemp from non-Florida climate and growing conditions
Hemp Practitioner (not cultivation) (*n* = 3)	Quality assurance, vertical integration and sourcing own raw material	Focus on quality and boutique uses (pet or personal healthcare)
General Public, Beginning Farmer (*n* = 5)	General interest in hemp, legalization as opportunity, personal connection to crop	Limited formal farming experience or training, small-scale production with vertical integration, in some cases non-commercial

### Established farmer or agribusiness

As the first profile and largest share of the group, a cohort of experienced farmers and agribusinesses from Florida (*N* = 5) or outside Florida (*N* = 2) stood out. If originating from outside Florida, these farmers were expanding into Florida to diversify their production region and benefit from the potential for year-round production. If originating from within Florida, they were seeking to diversify their crop portfolio, replace lost acreage due to the decline of citrus production or overcome limited growth potential with their existing crops (“We try out new stuff [new crops] constantly, and see what works”). Farmers described hemp as a novel and exciting crop that also held potential to interest and motivate younger generations in the farming operation (“We have been growing the same [ornamental flowers] for decades, my son wants to try something new”). This was brought up as an important factor for family business succession planning and breaking loose from established routines in their respective niche markets.

The established farmers and agribusinesses, both from outside and inside Florida, possessed a wealth of knowledge and resources, reflecting years of practice and a deep understanding of farming methodologies. Hemp farmers originating from outside Florida described more extensive experience with general hemp cultivation but faced challenges in adapting to local Florida growing conditions. Hemp farmers originating from Florida had more experience with the intricacies of farming in Florida but had no or significantly less experience with hemp cultivation.

Individuals sought information from a range of available sources, including insights from other growing regions in the US and abroad. Hemp production advice from established production regions (e.g., Europe or Asia) was at times described as more valuable than domestic advice and information, due to the longer production history (“the best information comes from outside Florida”). Growers tended to rely on research-based sources like university extension and the United States Department of Agriculture (USDA) more so than from industry suppliers. One grower commented that, “The vendors will sell you anything, but they don’t have experience in Florida”. Information sources for hemp were generally described as less robust than those for established crops like sugarcane or citrus. Attending national and international trade shows or conferences, as well as hiring external consultants, were common practices employed to quickly establish or resolve issues with hemp production in Florida.

The group of experienced farmers generally had dedicated personnel and resources to work and assess farming technology, which also included software and DSS. Drawing from experience with other crops or sophisticated production systems outside Florida, these farmers generally believed that a DSS can make or enable superior decision making (“The implementation of this [labor management software] tool was not easy, but it greatly improved our performance”). However, they also voiced concerns regarding the reliability and trust of underlying data for such models and decision support systems, given the limited history of cultivation in Florida. DSS were described as providing potentially valuable information, but requiring small-scale test runs or triangulation with other information (“I always say: trust, but also verify.“).

### Beginning farmers

The second largest share of interviewees (*N* = 5) were beginning farmers, often members from the general public venturing into farming for the first time. They went into hemp farming specifically due to their interest in the hemp plant and mostly produced on a smaller scale and focused on flower production for phytocannabinoids. Personal health concerns and positive experiences with hemp-derived products were mentioned as key factors for driving growers into hemp production. This led to a strong belief in the potential of locally grown hemp for natural remedies and community health. Growing and processing hemp aligns with the grower’s interest in holistic living and gardening. It is seen as an integral part of their lifestyle and home-based work and the community aspects was brought up repeatedly (“This plant has done so much for me and my family”).

Hemp production was often a part-time endeavor, due in part to the wide variation in profitability. Hemp production was generally described as a ‘passion’ project without immediate profit goals but still serves as a long-term vision. Growers faced obstacles typical of small-scale operations, such as the lack of specialized grow space. This led to self-built hybrid indoor/outdoor setups, a strong reliance on learning by doing, independent research, and cost-effective solutions. Small producers usually actively worked on vertical integration and development of their own marketing and sales channels for hemp products in their area.

Information sources varied widely but more often were focused on informal exchanges with other hemp enthusiasts via social media, as well as online forums and videos (“I just go on YouTube”). They described the whole range of possible farming challenges, although some challenges were more related to general farming in Florida instead of challenges specific to hemp due to limited previous farming experience. Farmers described that information sources from formal channels, such as cooperative extension programs, peer-reviewed articles, government agencies or company representatives were sometimes useful. In other cases, they were described as unsuitable for their small scale of production, or the growers were not aware of their availability.

Growers had less interest and stronger reservations concerning sophisticated technology and DSS as they often described their hemp production as artisanal or not industrial on purpose. Hemp production was often referred to as being an art and personal passion which requires manual labor and care of the plants. However, solutions like sensors, weather monitoring or automated irrigation systems were described as useful because production sites were often at some distance from their urban residence and the frequency of site visits was limited by their other professional activities. In this case, the technology could allow manual monitoring of general plant well-being from a distance and provide an indication if an in-person visit to the production site is needed.

### Hemp practitioner with non-agricultural background

The last distinct group that emerged from the interviews was that of hemp practitioners with no background in farming. This was, for example, a human or animal health care provider prescribing hemp-based products, who started producing their own hemp material to ensure high-quality supply or to meet specific product needs (“we needed this [hemp-derived product for small pet care] product, but just could not find it…so we started producing our own”). Hemp production activities were sometimes conducted in partnership with an experienced farmer. They had a strong focus on smaller scale but high-quality production and direct marketing through their existing contacts with customers.

The concepts of technology and decision support were well known from their main profession, since DSS in clinical settings or general healthcare have a long history and are widely used (Cresswell et al. 2012). Significant investments in hardware and software for automated production facilities and use of sophisticated DSS were described as solutions for the future or larger wholesale market growers. Their production was limited to their existing customer base and would not justify these expenses, although they expressed interest in more “what-if” scenario analysis tools that could support initial assessment of hemp production potentials (“Do we need 100 plants or a whole acre [4047 m^2^, thousand to thousands of plants depending on production type]?”).

### Hemp production challenges

**Fig. 5:**
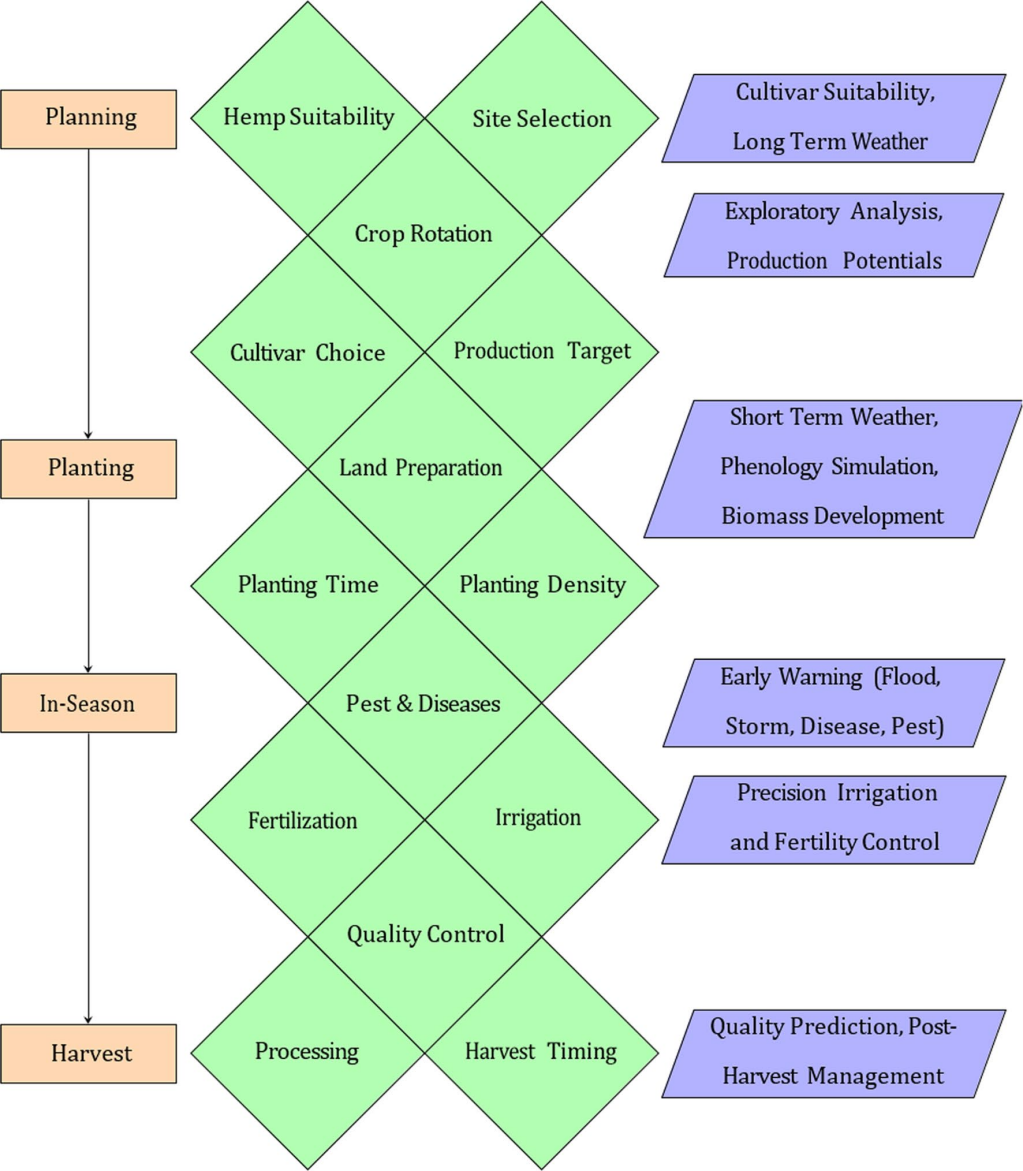
Overview of hemp production decision timeline derived from grower interviews. Rectangle shapes represent the phases of a production cycle, diamond shapes the decisions and trapezoid shapes the decision support opportunities. (source: main author)

**Fig. 6:**
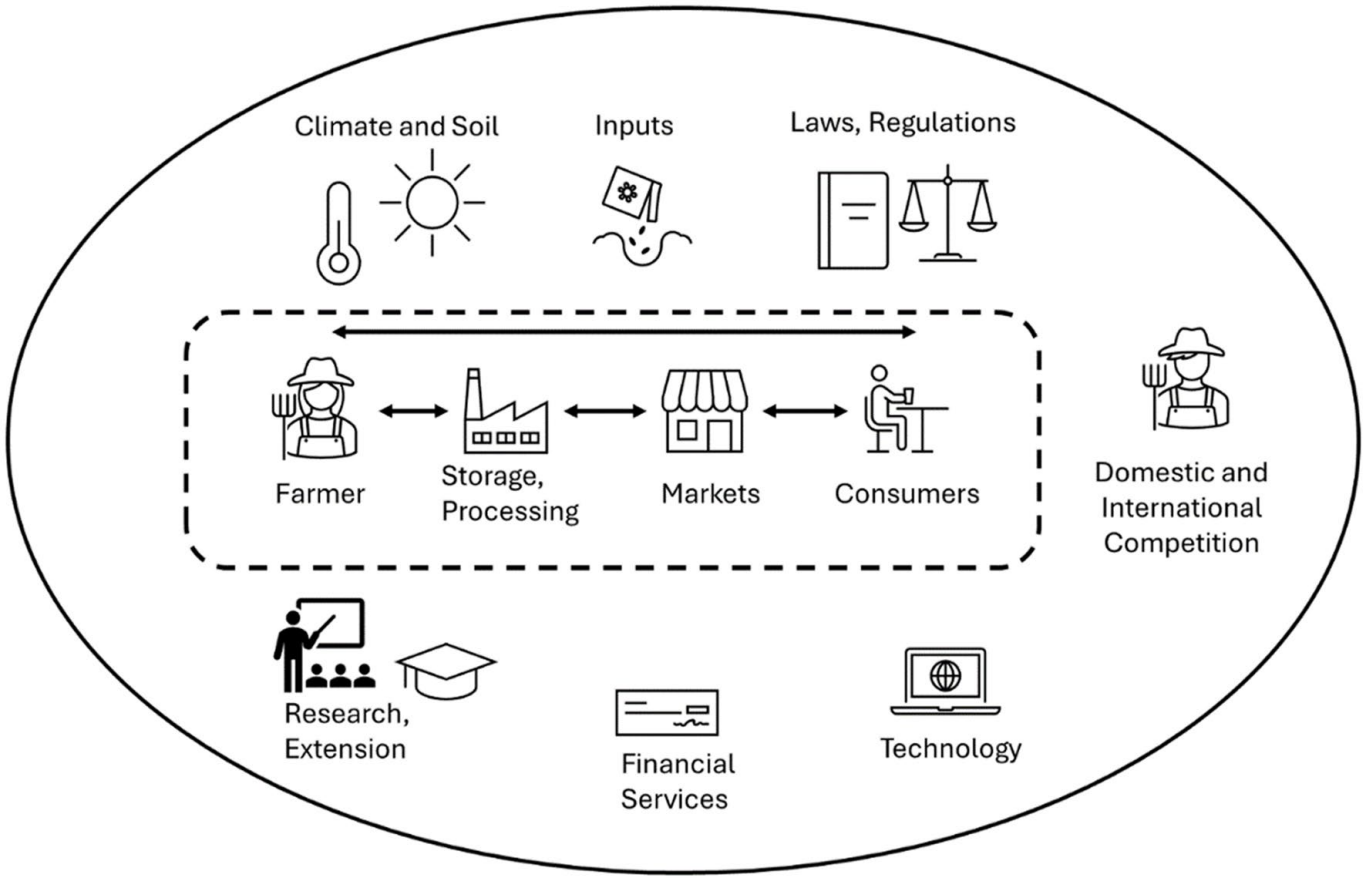
Landscape of hemp production described by farmers in Florida, with production, marketing and surrounding socio-economic and regulatory aspects. (source: main author)

Throughout the interviews, a series of agronomic challenges and decisions that occurred during a production cycle were identified (Fig. 5). Beyond agronomic challenges and knowledge gaps, regulatory uncertainty, infrastructure limitations and stigma were described as challenges for further growth and development of the hemp industry in Florida (Fig. 6). Although commercial hemp production is now legal with a permit, the regulatory framework surrounding its cultivation, processing and marketing is continuously evolving. Farmers must navigate frequently changing state and federal regulations, which creates uncertainty and can complicate business planning.

Florida lacks established infrastructure for hemp processing, particularly fiber processing, which presents a bottleneck in the supply chain. Growers face difficulty finding facilities to process their harvested hemp into value-added products, hindering the industry’s growth (“We couldn’t process the harvest that year, we lost most of it… and did not try again”). The demand for hemp-based fibers, CBD, and other products is generally growing (Expert Market Research 2024), but remains on a small level compared to other commodity crops. The overall market for raw material and hemp-derived products is therefore still developing and rather unstable (“It’s like the Wild West”). Florida-grown hemp must compete with products from other states and international markets, which creates challenges for market access and price stabilization. Despite regulatory changes, lingering stigma and misconceptions about hemp as a crop remain. This created obstacles for some farmers in securing financing, finding buyers, and obtaining the necessary licenses and permits.

### Barriers and opportunities for Model-based decision support

General barriers to adoption of DSS related to cost, applicability and trust in DSS. Many software-based DSS come with fixed or minimum prices, which make them inaccessible to the small hemp growers at an early stage of hemp cultivation (“it costs the same for 10 or 10,000 plants”). Beyond the direct cost of a DSS for hardware or software, the mental resources required to setup or engage with a DSS were also discussed. Smaller or single-individual farming operations mentioned being too occupied with production, processing, and marketing to learn or apply a specific DSS. Larger operations tended to have dedicated staff for machinery and technology who could study DSS or other solutions and their applicability to the overall operation (“I have a sensor guy who helps us look at this”).

In general, farmers mentioned limited trust in solutions that were developed using datasets from other growing locations such as California, Colorado or abroad. They articulated that these tools might not be suitable in the unique Florida environment and would want to see a validation or trial with local data first. Specifically critical decisions, e.g. the cannabinoid concentration that has legal implications, were of high interest but also low trust since they come with a high risk.

However, they expressed trust and belief in a general ‘superiority’ of DSS, if the underlying models are developed with sufficient local data (“…the AI can just see things we can’t see…”). An important line of thought emerged on the value and usefulness of DSS and model-based recommendations. Providing raw data and information in itself was described as of limited use compared to providing a specific solution to an agronomic problem or laying out a (model-based) pathway to achieve a certain production goal (“don’t give me more data or issues, show me a solution…”). The value of a DSS can be communicated through a direct return on investment (“% cost saving”) but also by preventing risks and losses (“It would have been good to try out some ideas virtually in a software first, instead of in the field”). Negative experiences with agronomic challenges or losses (“the whole plot was infested with fungus overnight…we were surprised, and a warning or prediction could have been useful”) were described as leading to increased interest in DSS to help manage these challenges.

Some closing conversations were centered around the human vs. computer aspects. Digital tools were described as preferred for their clarity, less biased information (“compared to sales representatives”) and lack of distractions (“the tool gives you the specific information, and just that”). However, interviewees also acknowledged the value of personal interactions for uncovering additional insights (“personal conversations can go off tangents, a lot of that extra is irrelevant…but some of it is very good”).

**Fig. 7:**
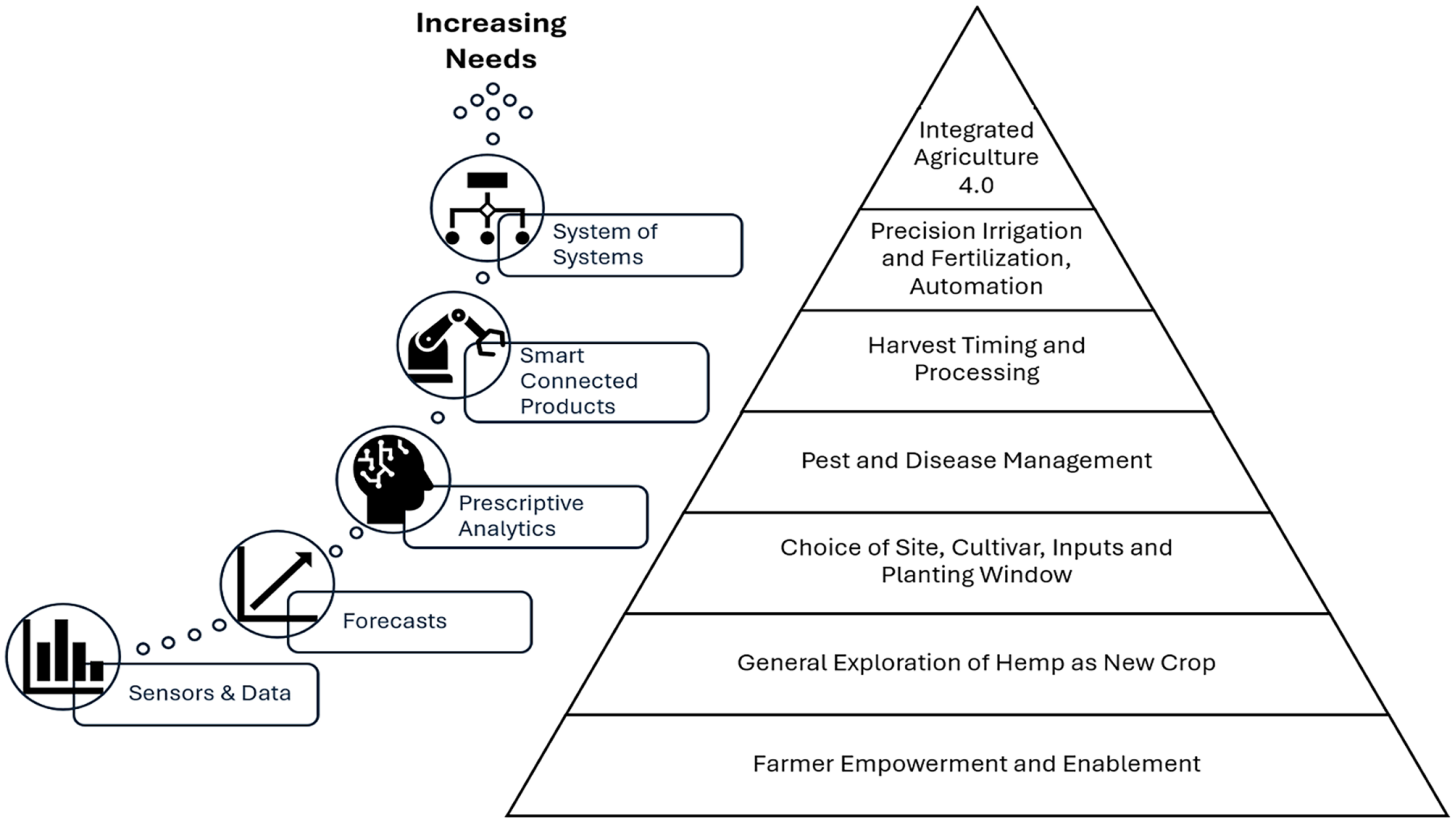
Hierarchy of decision support needs of a hemp grower, and related technologies that can support these decisions. (source: main author)

This leads to a ‘Hierarchy of Decision Support Needs’ (Fig. 7) derived from the interviews, where different agronomic challenges, farmer needs and technologies are related. Some of the foundational issues such as regulation, markets and stigma around hemp production in Florida are likely not addressable by agronomic DSS. Foundational agronomic questions such as the suitability of the crop to a given climate, potential production under different planting date or density scenarios could be answered by data, forecasts and models.

More advanced agronomic challenges in line with precision agriculture (e.g. pest management and harvest timing for quality management) require more sophisticated models, prescriptive analytics and deeper understanding of the system. Fully integrated ‘Agriculture 4.0’ production requires additional degrees of automation and communication between different aspects of the production system. This was often described as out of reach for still developing hemp production setups in Florida (“we use aerial imagery and auto-steering for out other crops, but with hemp we are still busy finding the right cultivars”).

### Implications

The interviews highlighted the need for localized DSS that are tested and validated to build trust. Hemp growers in Florida require DSS tailored to their unique environmental conditions that address specific challenges (e.g., pest management, cultivar choice, planting and harvest timing). Generic tools developed in other locations were met with skepticism. Nevertheless, the potential for DSS to prevent losses from pests, disease, or incorrect planting times could encourage adoption and support a general shift towards precision agriculture. However, more sophisticated DSS incorporating models and prescriptive analytics would be needed in the future to pave the way for ‘Agriculture 4.0’ approaches in hemp production. Looking at business and farming operations, cost-effective solutions are required. DSS for smaller hemp growers need to be affordable and easy to use, while larger agribusinesses are more likely to adopt DSS due to available resources for technology assessment and implementation. In both cases, they need to showcase a clear return of investment, either through cost savings or yield improvements although qualitative benefits such as peace of mind or increased knowledge are valuable.

DSS were discussed as an important component of risk assessment, as they can help farmers make informed choices, mitigating risks associated with crop failure, regulatory compliance, and market fluctuations. Besides the scientific capabilities of DSS, farmer education and training programs should focus on the value of DSS and how to interpret their recommendations effectively. This underlines the importance of cooperative extension programs and the extension agent as a potential intermediary between DSS developer and end user (Ayre et al. 2019; Eastwood et al. 2019). Educational materials should highlight the importance of using locally sourced data for model validation and trust-building, which connects to the need for continued investment in research to collect comprehensive, region-specific data for Florida hemp cultivation. Subsidies and grants could incentivize the development and adoption of affordable, locally adapted DSS for small-scale growers, which should also include data sharing among farmers and researchers to improve DSS development and adaptation. Recognizing that farmers’ objectives can vary significantly, DSS design should ideally accommodate this heterogeneity. Future DSS development should therefore consider features that allow for customization based on differing priorities or be designed with the advisor as a key end-user who facilitates this tailoring. Despite the limited hemp cultivation in Florida in recent years, there is a growing market opportunity for companies to develop specialized DSS for cultivation of hemp and other crops, and an opportunity to provide overarching services that help farmers collect, analyze, and interpret data.

As a concrete example for future research, a tool situated for decision in the pre-planting to planting stage was identified as a first manageable application for DSS. The tool can assist in the exploratory analysis of the hemp cropping system and compare management options such as cultivar choice or planting date and density. A tool of this type has potential to prevent some of the discussed mistakes identified, such as planting too early or too late, unsuitable cultivars, or complications with timing of the overall crop rotation. Furthermore, it can provide an initial assessment of potential productivity and production cycles for a given location.

### Limitations

While this study sheds light on critical issues facing industrial hemp farmers regarding DSS adoption, there are limitations to consider. Our focus was primarily on a limited sample size within Florida and provides location, crop and context specific findings. Validity and reliability, also termed trustworthiness or rigorousness, is an important concept in social science research (Drost 2011). The use of software for the thematic analysis and coding process, such as NVivo, has been extensively discussed by Welsh (2002), and we assessed both manual and electronic methods for this study. Statistical indicators for validity and reliability are not applicable in this study because no quantitative or demographic information was collected in a structured manner.

Testing procedures such as split-half approach or test-retest reliability were not employed due to the small sample size available. We acknowledge that the research might have limited external validity beyond the specific target population of hemp producers in Florida. To the extent possible, results and conclusions from the interviews were triangulated or contrasted to findings from the literature, both related to hemp production but also more broadly to the topic of decision support systems in agriculture.

The underlying approach of this study was focused on process-based and biophysical model-driven DSS in line with the overarching topic of the extended research project. This gave limited consideration to other modeling approaches, such as conceptual models, economic models, agent-based model, knowledge-based models or expert systems.

### Future research

Future research should include additional interviews with a larger group of farmers with different sociodemographic backgrounds, types of production and experiences, including farmers from outside Florida. The anticipated larger volume of data could be evaluated using novel AI-based analytical techniques as outlined by Jiang et al. (2021). While this study focused on qualitative interviews, incorporating quantitative data collection methods, such as surveys or economic modeling, could provide a more comprehensive understanding of the demographics and economics of hemp farming in Florida. Conducting a longitudinal study that follows a cohort of hemp farmers over several growing seasons could reveal how their experiences, challenges, and strategies evolve over time. This would shed light on the long-term sustainability and adaptability of hemp farming in Florida’s dynamic agricultural landscape.

Comparing the experiences and challenges of hemp farmers in Florida with those in other states or countries could illuminate unique regulatory, climatic, or market factors influencing the industry’s development in different regions. This would offer valuable insights for policymakers and industry stakeholders seeking to optimize hemp cultivation practices and policies. Analyzing the impact of existing and proposed policies on hemp farming or general agricultural technology in Florida could identify potential barriers or incentives for farmers. Additionally, cultural and ideological factors influencing these farmers warrant further study. Lastly, deeper analysis of the psychological and behavioral factors driving innovation decisions among hemp farmers is needed.

## Conclusions

This study demonstrates the need to understand the concerns specifically faced by Florida’s industrial hemp farmers because these factors heavily influence their attitude or decision to adopt DSS. Previous studies have often overlooked the unique needs of this sector. Our research intends to address this and provide actionable guidance for policymakers and stakeholders seeking to support the growth of Florida’s hemp industry.

Farmers, especially those within the emerging industrial hemp market, are driven to improve productivity and profitability. However, concerns about the financial burdens of new technologies, their operational complexity, and potential compromises in accuracy in this new environment can hinder adoption. Florida’s hemp farmers experience substantial barriers to profitable production and are nonetheless receptive to tailored solutions that minimize production and technological concerns. The type of tool and degree of digitization or technology involved for useful application varies by farmer type. The development of a tool aimed at pre-planting decision, for exploratory analysis, was derived from the interviews for future work.

Despite the overarching hurdles and thinking beyond technology and DSS, most interviewees reiterated Florida’s potential as a significant hemp producer. The state’s year-round growing season, agricultural expertise, and proximity to major markets were mentioned as distinct advantages. They expressed support and hope for ongoing research programs focused on developing hemp varieties specifically adapted to Florida’s conditions and establishing best cultivation practices supported by different degrees of technology. Streamlining the policy and regulatory environment were mentioned as important to strengthen the foundation for the industry and markets.

## Supplementary Information

Below is the link to the electronic supplementary material.

Supplementary Material 1

## Data Availability

The full interview guide and recruitment materials are presented in Additional Files A, B and C. The complete interview transcripts and notes generated and/or analysed during the current study are not publicly available due to the anonymity and confidentiality promised during the interview process. Interview notes can be made available from the corresponding author on reasonable request.

## Abbreviations

AI: Artificial Intelligence
CBD: Cannabidiol
DSS: Decision Support System
IRB: Institutional Review Board
TAM: Technology Acceptance Model
