# Supplementary material for: Assessment of the knowledge landscape, information needs and attitude towards decision support systems among hemp farmers in Florida
Source: J Cannabis Res. 2025 Aug 20;7:62. doi: 10.1186/s42238-025-00318-3 (PMC12366241; doi:10.1186/s42238-025-00318-3)
Supplement: Supplementary file 1 — Supplementary Material 1 [file 42238_2025_318_MOESM1_ESM.docx]

# Additional Files

## Additional Files A: Interview Recruitment Flyer


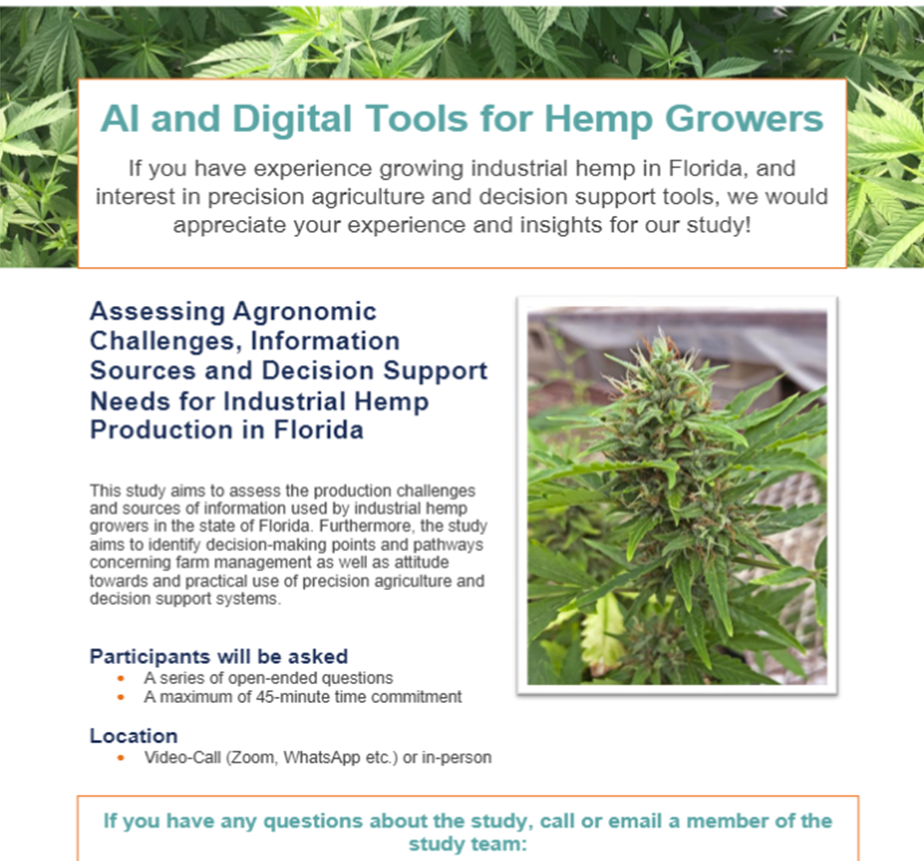


## Additional Files B: Informed Consent Form

***Research Participant Informed Consent Form***

Please read this document carefully before you decide to participate in this research study. **Your participation is voluntary, and you can decline to participate, or withdraw consent at any time, with no consequences**.

Study Title:

Assessing Agronomic Challenges, Information Sources and Decision Support Needs for Industrial Hemp Production in Florida

Person conducting the research:

(removed identifying information)

Purpose of the research study:

The purpose of this study is to assess the production challenges and sources of information used by industrial hemp growers in the state of Florida. Furthermore, the study aims to identify decision-making points and pathways concerning farm management as well as attitude towards and practical use of precision agriculture and decision support systems.

What you will be asked to do in the study:

I will ask you a number of questions about various topics of interest in my research.

Time required:

The expected time required will be one single session lasting no more than 45 minutes.

Risks and benefits:

There are no risks or discomforts anticipated. There are no direct benefits of participation for you.

Confidentiality:

Your identity will be kept confidential. We will not use your name in any type of publication or the final report of the results of this study. We will record your responses if you consent and only use the recording to create a transcript for later review. Your name will be replaced with a pseudonym and not be associated with responses once the recordings are transcribed and erased. Data will be stored on a computer at the University of Florida using the Microsoft OneDrive file hosting service. There is a minimal risk that security of any online data may be breached, but since (1) any identifying information is stored separately from your responses, and (2) Microsoft OneDrive uses several layers of encryption and firewalls, it is highly unlikely that a security breach of the online data will result in any adverse consequence for you.

Compensation:

No compensation will be provided.

May the researcher(s) benefit from the research?

We may benefit professionally if the results of the study are presented at meetings or in scientific journals.

Withdrawal from the study:

You are free to withdraw your consent and to stop participating in this study at any time without consequence. You can decline to answer any question you don’t wish to answer.

If you withdraw, will your information be used or discarded?

The information will be discarded.

Can the researcher(s) withdraw you from the study? If so, on what basis?

No, the researcher(s) cannot withdraw you from the study.

If you wish to discuss the information above or any discomforts you may experience, please ask questions now or contact one of the research team members listed at the top of this form.

If you have any questions regarding your rights as a research subject, please contact

(removed identifying information)

**Agreement:**

Do you voluntarily consent to participate in this study? If so, we will proceed.

## Additional Files C: Interview Guide

**Semi-Structured Interview Framework for IRB: IRB202202398
Assessing Information Sources and Decision Support Needs for Industrial Hemp Production in Florida**

Note: Questions in bold blue letters are considered key questions, remaining questions might be omitted under time constraints. Text in [] serves as exemplary transition phrases. “Probe:…” remarks serve as memory aid for potential follow-up or specification of the responses.

**Section 1 – Introduction**

***[****Greeting and general intro, if not done already during introduction and informed consent****]***

**1.1 What brought you into hemp farming and made you explore this new crop?**

1.2 Can you tell me about your general farm operation?

1.3 Are you growing exclusively hemp or also other crops?

1.4 When did you start growing hemp?

1.5 What markets are you targeting?

- Probe: fiber vs. floral, processing, wholesale, own label etc.

**Section 2 – Agronomic Challenges with Hemp**

*[I have a few questions about your production challenges with hemp…]*

2.1 You just harvested, how was your season?

- Probe: What was your biggest challenge?
  - Have you had successful crops this/previous seasons?
  - What does “having a good season” mean to you? Yield? Prices?

**2.2 Walk me through the most critical farming decision in your year…**

- **Starting with season planning, obtaining seeds, planting, in-season management to harvest**
- **Probe top 3: fertilizer management, site selection, cultivar choice and planting rate, irrigation, pest management, crop rotation, challenges beyond production such as legal issues or something else**

2.3 Do you employ precision agriculture technology (machinery, GPS, sensors, software), on your farm to help with management and production?

- Probe: Is this technology unique for hemp or the same for all crops? Do you use other technologies for non-hemp crops but not for hemp?

2.4 Do you follow or practice certain Best Management Practices (BMP’s)?

- Probe: Did they have any effect on your farm or environment?
- Definition BMP: Florida Department of Agriculture and Consumer Services’ Best Management
  for…Nutrient management, irrigation management, water resource protection

2.5 If following any BMP: Can you outline how you ended up adopting and implementing [xzy]?

- Probe: Did (digital) technology played a role in that or not?

**Section 3 – Perceptions and Use of Information Sources**

*[I would like to learn more about your perception and use of various information sources…]*

3.1 What sources of information are most important for your farm management?

- Probe – Source: University extension, commercial company representatives, private consultants, other farmers, personal experience? [give piece of paper to rank]
- Probe – Channel: TV/radio news, trade shows and conferences, conversations, websites, software, or apps
- Probe: name of news outlet or name of apps if specific one identified

3.2 When starting to explore hemp production, did you experience any constraints in finding information?

- Probe: lack of possible knowledge exchange with neighbors? More open towards external information?

3.3 When planning out your hemp production for the year, how do weather extremes influence your management approach or decision making?

**Section 4 – Digital Tools and Decision Support Systems**

*[Many predictive tools and precision agriculture technologies promise to help you grow more efficiently and sustainably, while providing you insights that can facilitate your own decision making. I would like to discuss your experience and opinion on digital tools for farm management…]*

4.1 How do you feel about using digital tools, such as apps or software, to manage your farming operations?

- Probe: are you using any specific tool/software?

**4.2 You previously [Question 2.2] indicated x, y, and z as most critical farming decisions. Imagine a tool that would provide a forecast or recommendation regarding this decision, e.g., [a recommended fertilizer application based on crop rotation and soil type]. What do you think about the usefulness and practicalities of such as tool?**

4.3 Beyond the specific hemp farming decisions, could you describe the last time you used an app/software/tool to guide your decision making in other areas?

- Probe: What kind of decision were you making and how did the tool assist you?

4.4 Do you recall a situation where a recommendation (from a digital tool or non-digital) was contrary to your own experience or opinion?

- Probe: Can you describe the situation and course of action you took?

4.5 Are there other production tools that growers should have available or must use these days to stay competitive?

**Section 5 – Closing and General Demographics**

*[We discussed some challenges with precision agriculture and decision support systems for farm management…]*

**5.1 Thinking about your own farm’s balance between hemp farming, general farming, and other activities: Are you and your enterprise “where you want to be”?**

- Probe: Do you see changes or the need for changes to your farm and farming operations in the future? (Probe: shifting markets, selling, or buying land, adopting new technologies, abandoning hemp again)

5.2 Reflecting on your previous answer, do you think your experiences are specific to hemp or also apply to general farming in Florida?

5.3 Can you share key indicators about your farm such as?

- Acreage
- Typical rotation if applicable, role of hemp
- % Income from hemp
- Number of full-time and seasonal employees
- Gender, age, ethnicity

5.4 Potential Follow up: you mentioned …., can you explain why/how/elaborate?

5.5 Closing questions: We talked about [xyz], tell me: what is missing...? Does it really matter? Was this important for you to talk about?
